# X-ray Structures of the Signal Recognition Particle Receptor Reveal Targeting Cycle Intermediates

**DOI:** 10.1371/journal.pone.0000607

**Published:** 2007-07-11

**Authors:** Christopher L. Reyes, Earl Rutenber, Peter Walter, Robert M. Stroud

**Affiliations:** 1 Graduate Group in Biophysics, Department of Biochemistry and Biophysics, University of California at San Francisco, San Francisco, California, United States of America; 2 Howard Hughes Medical Institute, Department of Biochemistry and Biophysics, University of California at San Francisco, San Francisco, California, United States of America; 3 Department of Biochemistry and Biophysics, University of California at San Francisco, San Francisco, California, United States of America; Massachusetts Institute of Technology, United States of America

## Abstract

The signal recognition particle (SRP) and its conjugate receptor (SR) mediate cotranslational targeting of a subclass of proteins destined for secretion to the endoplasmic reticulum membrane in eukaryotes or to the plasma membrane in prokaryotes. Conserved active site residues in the GTPase domains of both SRP and SR mediate discrete conformational changes during formation and dissociation of the SRP·SR complex. Here, we describe structures of the prokaryotic SR, FtsY, as an apo protein and in two different complexes with a non-hydrolysable GTP analog (GMPPNP). These structures reveal intermediate conformations of FtsY containing GMPPNP and explain how the conserved active site residues position the nucleotide into a non-catalytic conformation. The basis for the lower specificity of binding of nucleotide in FtsY prior to heterodimerization with the SRP conjugate Ffh is also shown. We propose that these structural changes represent discrete conformational states assumed by FtsY during targeting complex formation and dissociation.

## Introduction

The functional core proteins of the signal recognition particle (SRP) and the SRP receptor (SR) (called Ffh and FtsY in bacteria) contain GTPase domains and form a distinct subfamily of GTPases. These GTPases mediate cotranslational targeting of secretory and membrane proteins to the endoplasmic reticulum (ER) membrane in eukaryotes or the plasma membrane in prokaryotes (for a review, see [1]). The classical GTPase motifs I-IV (also referred to as G1-G4) [2] are present in both SRP GTPases and show marked conservation with p21Ras. Present in and unique to SRP and SR are four additional elements, the insertion box domain (IBD), the closing loop, the ‘DARGG’ motif and the ‘ALLEADV’ motif. These elements contain essential structural functionality for SRP GTPases (for a review, see [3]). In contrast to the ‘classical’ model of GTPase regulation by external factors, SRP GTPases interact directly to reciprocally stimulate GTP hydrolysis and neither requires an exchange factor for nucleotide release [4]–[6]. The SRP family of GTPases thus provides a unique variation to the ‘classical’ GTPase model and the elucidation of the underlying mechanisms involved in regulating the targeting reaction is at the core of current structural and biochemical studies.

A family of crystal structures of prokaryotic SRP GTPases illustrates this unique mechanism of activation. In particular, apo structures of Ffh-NG, a truncated version of the prokaryotic SRP core protein containing the amino- and GTPase domains, and FtsY, the SR protein, show the stabilization of an “open” state through interactions of the GTPase and SRP conserved sequence motif residues [7], [8]. “Open” state conformations in the presence of either bound nonhydrolyzable GTP substrate analog guanine 5′-imidotriphosphate (GMPPNP) or product GDP have been shown for Ffh-NG [9], [10]. Similarly a structure of FtsY with the product, GDP has been obtained [11]. In this structure, the GDP is coordinated with canonical binding interactions and reveals the importance of the C-terminal helix in the NG packing interface. In addition, three additional apo structures of FtsY have been solved and show distinct properties from FtsY in complex with Ffh [12]–[14]. Low measured intrinsic GTPase activities of Ffh and of FtsY (0.09 min^−1^, 0.01 min^−1^ ) [15] imply that proteins bind GTP and remain in “open” conformational states. Low specificity for nucleotide in monomeric FtsY has also been shown and further suggests a novel structural regulation of GTPase activity for FtsY [16].

The structures of the FtsY·Ffh-NG complex in presence of the non-hydrolyzable substrate analog GMPPCP [17], [18], GMPPNP [19], or GDP:AlF_4_ [20] show the formation of a composite, active site sequestered from solvent, through the catalytic interactions between the classical GTPase and SRP specific conserved elements from both GTPases and the bound nucleotides. Nucleotide hydrolysis in each active site drives dissociation of the SRP·SRP receptor complex, allowing the SRP and SRP receptor components to be recycled [21], [22]. Interestingly, the FtsY·Ffh-NG complex structures also suggest that *(i)* the structures observed represent a ground state of the GTP hydrolysis reaction, and *(ii)* that additional conformational changes in the active site are necessary to progress to the transition state. Recently, mutational studies of FtsY have revealed that site-specific mutations can modulate discrete conformational changes during Ffh·FtsY complex formation [23]. These specific conformational states involve the sequential activation of the Ffh and FtsY active sites after binding of GTP and during the formation of the targeting complex.

Here we report the structures of two conformations of FtsY in complex with the substrate analogue GMPPNP. These structures reveal two novel active site architectures for SR GTPase-nucleotide complexes. These structures, along with the structure of apo FtsY and FtsY in the Ffh-NG·FtsY complex, can be interpreted as a series of discrete conformational states along the pathway of step-wise activation of the SRP GTPases during the formation of the SRP·SR complex and provide structural explanations for the biochemical differences observed for FtsY along the targeting cycle.

## Results

### Structures of FtsY·GMPPNP complex

**Figure 1:**
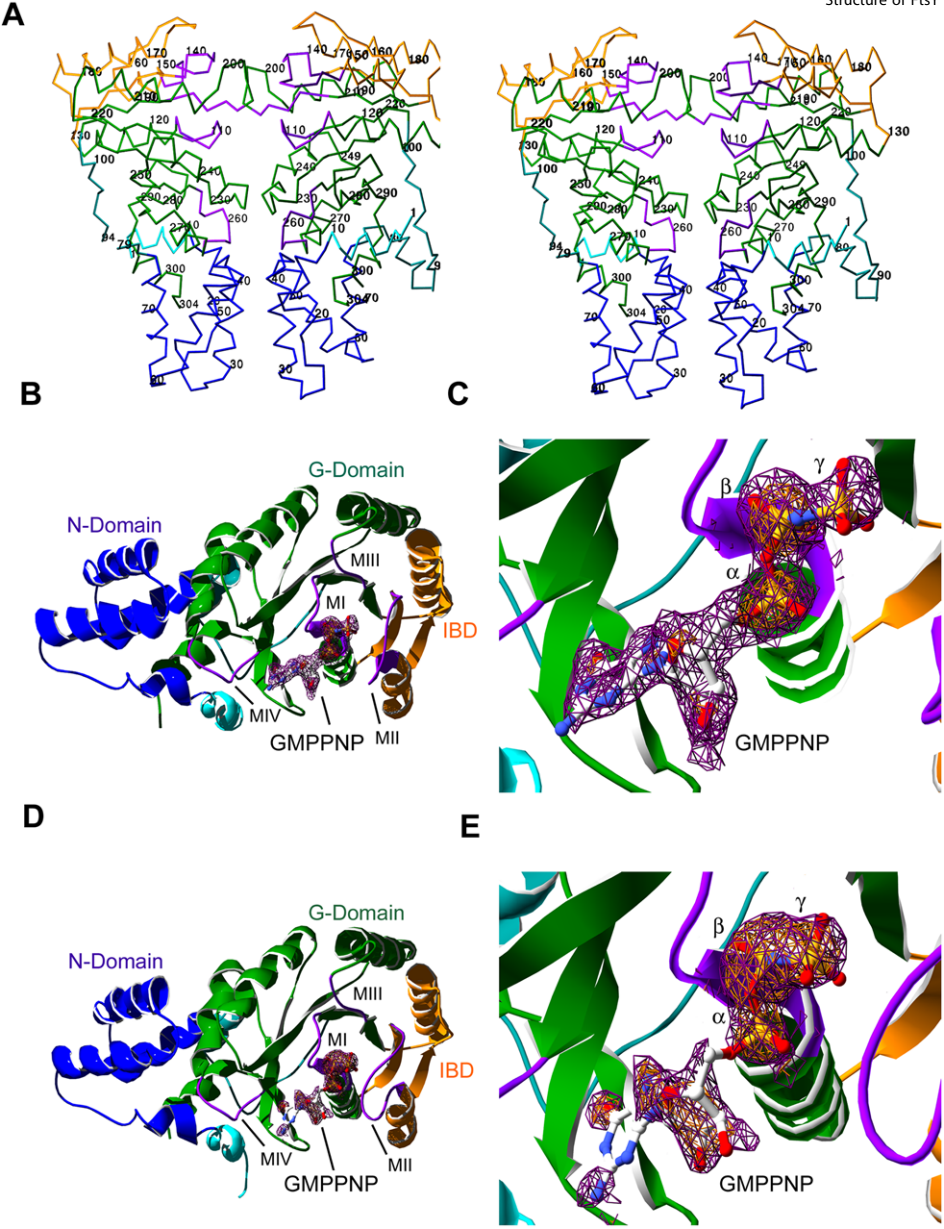
Structure of FtsY apo/GMPPNP dimer from *Thermus aquaticus.* (A) Stereo view of the Cα backbone trace of FtsY apo/GMPPNP dimer (nucleotide not shown). The structure of FtsY is comprised of the N-terminal helix (residues 1–10), shown in cyan; the N-domain in blue; the G-domain in green; the insertion box domain (IBD) in gold and the conserved GTPase motifs (MI, MII, MII and MIV) in purple. (B) A ribbon diagram of the structure of FtsY in complex with non-hydrolyzable substrate analog GMPPNP crystallized in the presence of MgCl_2_. No evidence for a coordinated Mg^2+^ ion was observed similar to the structure of GDP-bound FtsY [10]. (C) A simulated annealing 2Fo–Fc omit map, contoured at 1σ, is shown. The bound GMPPNP was omitted. (D) Ribbon diagram of the structure of FtsY in complex with GMPPNP crystallized in the absence of MgCl_2_. (E) A simulated annealing 2Fo–Fc omit map, contoured at 1σ with bound GMPPNP omitted. In the absence of Mg^2+^, the guanine and ribose moieties of the bound GMPPNP are less ordered than in the presence of MgCl_2_ (C).

Two crystal forms of FtsY from *Thermus aquaticus* (*T. aquaticus)* were crystallized and structures determined. Crystal form one initially grew out of a purified complex of FtsY, full length Ffh from *T. aquaticus*, GMPPNP and Mg^2+^. Subsequent crystals were grown in the absence of Ffh. The structure of this form was determined to 2.2 Å resolution and contained a single non-crystallographic (NCS) two-fold related dimer per asymmetric unit with one monomer in apo-FtsY and the other FtsY monomer bound with GMPPNP (Figure 1A, 1B, 1C). A second form containing the apo/GMPPNP bound dimer was also initially crystallized from the purified FtsY▪Ffh▪GMPPNP complex in the absence of Mg^2+^ in different crystallization conditions from crystal form one (Figure 1D, E) (Table 1). In addition, apo crystals of FtsY pre-derivatized with chloromercurial nitrophenol (CMNP) were grown and solved by multiple wavelength anomalous diffraction (MAD) and used as a molecular replacement model for the apo/GMPPNP dimer forms. For the purposes of this paper, we will refer to the GMPPNP bound monomer from crystal form one as F1 and from crystal form two as F2.

**Table 1:** Data collection, phasing and refinement statistics

	Crystal form I-F1	Crystal form II-F2	FtsY-CMNP
*Data processing*			
Source (wavelength, Å)	SSRL 7-1 (λ = 1.08)	SSRL 9-1 (λ = 0.98)	ALS 5.0.2
			(λ1 = 1.006, λ2 = 1.009, λ3 = 0.993)
Space group	P2_1_2_1_2_1_	P2_1_2_1_2_1_	P2_1_2_1_2_1_
Unit cell, Å	a = 63.40 b = 96.28 c = 99.10	a = 63.84 b = 97.30 c = 99.28	a = 63.62 b = 96.68 c = 99.56
Resolution, Å	2.2	2.3	1.8
Measured reflections	116,338	78,582	682,554-λ1
			588,438-λ2
			553,320-λ3
Independent reflections	29,084	25,423	57,696-λ1
			57,434-λ2
			57,458-λ3
*R*_sym_ a, %	10.1	6.3	7.0-λ1
			7.7-λ2
			8.1-λ3
Completeness, %	93.8 (94.0)†	91.9 (90.3)†	100.0 (99.9)†-λ1
			99.8 (99.8)†-λ2
			99.7 (99.7)†-λ3
<I/σI>	6.6	11.5	7.8-λ1
			6.5-λ2
			5.6-λ3
*Refinement statistics*			
*R*_cryst_ b, %	20.3	20.8	22.3
*R*_free_ c, %	27.4	26.9	24.7
Rmsd^d^ bond lengths, Å	0.02	0.03	0.02
Rmsd^d^ bond angles, deg	1.69	2.19	1.56
<B>, Å^2^			
protein	16.8	22.0	14.5
GMPPNP	39.7	58.8	n/a
water molecules	32.7	41.6	39.2
PDB code	2Q9C	2Q9B	2Q9A

† Numbers in parentheses are the high-resolution bin.

a *R*_sym_ = Σ|I-<I>|Σ<I>, where I is the measured intensity of each reflection, and <I> is the intensity averaged from symmetry equivalents.

b *R*_cryst_ = Σ|F_o_-F_c_|/Σ|F_c_|, where F_o_ and F_c_ are observed and calculated structure factors, respectively.

c *R*_free _was calculated from a test set (8–10%) omitted from the refinement.

d rmsd, root mean square deviation.

**Figure 2:**
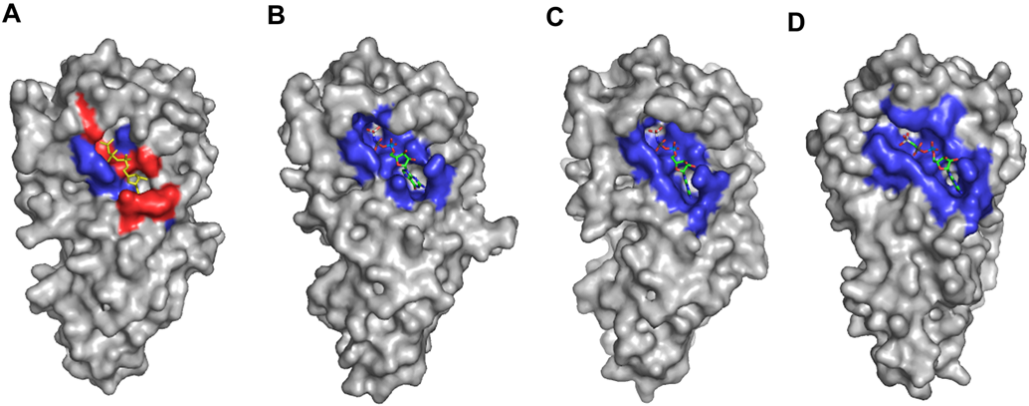
Adaptation of FtsY active site to nucleotide during targeting cycle. Residues in contact within 4 Å of nucleotide are shown in blue. Residues clashing with nucleotide position are shown in red (contacting distance ≤2.5Å). (A) Apo FtsY with docked GMPPNP (yellow) from FtsY - GMPPNP/Mg position. The active site is not formed to have extensive contacts with the putative position of nucleotide. The P-loop, motif III, motif IV and closing loop all contain residues that are in position to “clash” with the nucleotide. (B) FtsY in complex with GMPPNP (green cpk). The active site shows limited contacts (blue) with the nucleotide. The residues of the conserved motifs that were in position to clash with the nucleotide in the apo structure are now repositioned favorably to interact with the nucleotide. (C) FtsY in complex with GMPPNP (green cpk) in presence of Mg^2+ ^. The active site contacts are more extensive than in the complex with GMPPNP without Mg^2+.^ (D) FtsY in complex with Ffh and GMPPCP (green cpk). The active site pocket contains contacts that are more extensive than in the monomer FtsY structures.

The FtsY dimer is related to the FtsY·Ffh-NG complex dimer by a ∼20° rotation of the NCS two-fold. The apo monomer from crystal form one and crystal form two are nearly identical and show a relative shift in the NG interface compared to F1 and F2. The overall fold of FtsY observed for the various conformations, are nearly identical. Although the F1 crystal form of FtsY was co-crystallized with magnesium chloride, identification of an ordered magnesium ion in the structure is uncertain. An ordered water is observed coordinated with the γ-phosphate and four additional waters. This would be a likely candidate for a magnesium ion, except that the bonding distance is more than 0.5 Å greater than expected for a coordinated magnesium ion. Therefore, the molecule was modeled as water and no magnesium is present in the final model. It should be noted that a magnesium ion is also not observed in the structure of Ffh-NG bound with GMPPNP [10]. Present in F1 and F2 are the first 27 N-terminal residues not seen in the FtsY monomer of the FtsY·Ffh-NG complex; they are structured in a loose helical manner. Overall, the FtsY·GMPPNP structures and the apo structure demonstrate varying degrees of an oversized “open” conformation in comparison to the FtsY structure from the FtsY·Ffh-NG complex (Figure 2). The most significant differences between the active form (from Ftsy·Ffh-NG), the two GMPPNP bound forms and the apo form, are localized in the conserved sequence motifs.

### Nucleotide specificity is poor

**Figure 3:**
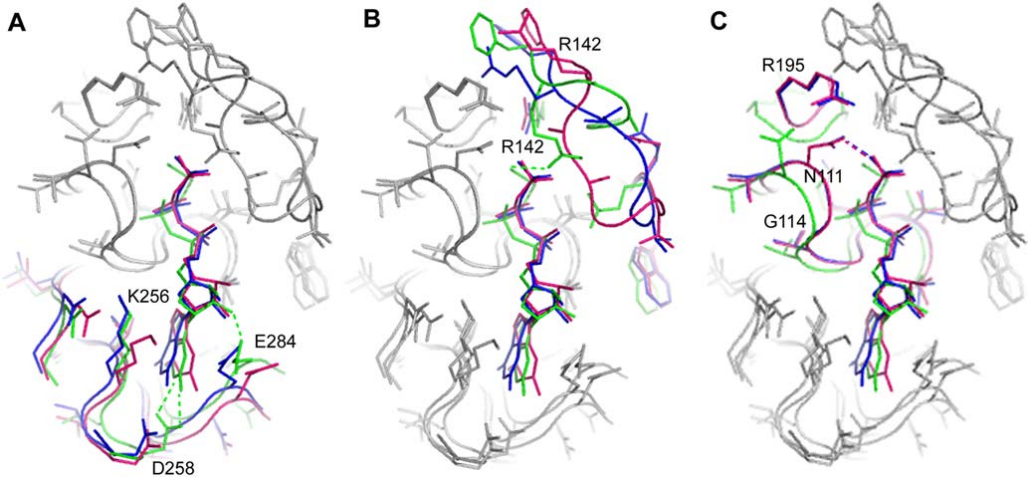
Structural adaptations in the active site upon nucleotide binding. (A) Asp258 is shown to progressively coordinate the nucleotide from the apo state to the Ffh-NG complex state. In F2 (magenta), the nucleotide is not coordinated by Asp258. In F1 (blue) the nucleotide is within weak coordinating distance to Asp258. In FtsY from the Ffh complex (green), Asp 258 coordinates the nucleotide (hydrogen bonds shown as dotted lines). (B) IBD residue Arg142 is shown to coordinate the magnesium ion and interact with the γ-phosphate in the complex form of FtsY (green). In F1 (blue) and F2(magenta), the ‘DTFRAGA’ motif unfolds and positions Arg142 out of the active site and away from interaction with the bound nucleotide. (C) In complex with Ffh-NG (green), the FtsY sidechains of Arg 195 and Asn111 are positioned out of the active site and away from the γ phosphate. In F1 (blue) and F2 (magenta), both Arg195 and Asn111 rotate towards the γ phosphate and the amino moiety of Asn111, now coordinating the γ phosphate in a non-canonical position.

The guanine base is coordinated by residues in motif IV which contains the conserved SRP GTPase ‘**TK**X**D**’ sequence motif. Aspartate 258 encodes specificity for the guanine base while lysine (Lys256) is involved in maintaining the active site cavity spacing through interactions with the P-loop and an aspartate in the α3 helix (Figure 3A). A concerted sequestering of the nucleotide is shown in the conformation of FtsY in complex with Ffh-NG: Asp258 coordinates the N1 and N2 amino groups of the guanine base at an average bond distance of 2.7 Å, the closing loop Glu284 packs against the ribose moiety of GMPPCP (2.6 Å), and Lys256 forms a bridging interaction with both the carbonyl oxygen of Gly112 of the P-loop at 2.8 Å and a 2.7 Å hydrogen bond with Asp229 of helix α3. In the F1 form with GMPPNP, Lys256 also forms a bridging hydrogen bond with the sidechain of Asp229. The interaction distance between the α3 helix Asp229 and motif IV Lys256 is on the same order as seen in the active conformation. However, the lysine is now at a distance of 3.6 Å from the P-loop. The effect of this loosening is a movement of motif IV away the P-loop and in turn away from the nucleotide such that Asp258 is now on average 3.4 Å from the amino groups of the guanine base. The closing loop also has retracted from the nucleotide, although Glu284 and the O3 of the ribose ring in GMPPNP are at the same interacting distance as in active FtsY as the glutamate extends away from the backbone. F2 also lacks the interaction between Lys258 and the P-loop (6.8 Å) and the distance from Asp258 to the amino groups extends even greater than in F1 to an average distance of 4.6 Å. Movement out of the active site by motif IV now decouples the interaction between the α3 helix Asp229 and motif IV Lys256 (now at a distance of 4.5 Å). In addition, the closing loop no longer packs against the nucleotide and Glu284 is 4.7 Å away from the ribose oxygen. Finally, in the ground state apo FtsY, the position of the guanine specifying Asp258 is further extended than in F1 or F2. Interestingly, the interaction distance between Lys256 and Asp229 is maintained but the interaction with the the P-loop is decreased by more than 1 Å. Overall, starting with the apo conformation and ending with the complex conformation, these interactions serve to organize a closure of the active site around the nucleotide to allow for increasing coordination of the guanine base by residues from motif IV; a closing which in turn is translated to the α3 helix.

### Non-canonical positions of γ-phosphate and active site residues

A comparison of the FtsY in complex with Ffh-NG, GMPPNP and apo structures reveal that the largest conformational differences localize to the SRP conserved sequence elements that coordinate the phosphate groups of the nucleotide. The Insertion Box Domain (IBD), a unique motif II sequence element contains two critical residues: Arg142 and Gln148. In complex with Ffh-NG, FtsY residue R142 interacts with γ-phosphate and Gln148 helps coordinates the magnesium ion (Figure 3B). A striking difference between this conformation of the IBD and the conformation in F1 and F2 is observed. In the GMPPNP bound forms, Arg142 has moved away from the active position in a greater than 5 Å movement accompanied by an unfolding and rearrangement of the ‘DTFRAGA’ sequence in the α1 helix of the IBD. This unfolding event also leads to a counterclockwise rotation (relative to the N to C axis of the α1a helix) of the Gln148 sidechain away from the active site and excludes any interaction with a magnesium ion. In apo FtsY, the IBD has re-structured and as a consequence Arg142 and Gln148 have returned to the active site although their positions differ from the active form. By superimposing bound nucleotide from the active form, F1 and F2, a clash in the van der Waals spacing between the apo position of this motif II arginine sidechain and the γ phosphate position of bound nucleotide is observed (not shown). In addition, Gln148 has rotated clockwise (from the same reference axis as above) greater than 2 Å from the active position to locate itself again out of the active site but in the opposite relative direction than in F1 and F2.

Binding of nucleotide is expected to confer certain conformational changes in the protein to both accommodate the nucleotide and to orient the active site moieties for proper hydrolysis. However, as also observed in Ffh-NG bound with GMPPNP, FtsY does not appear to be in a state that would allow for proper nucleotide hydrolysis. Lys115 is displaced from the active site cavity and does not interact with the phosphate groups of GMPPNP (Figure 3C). Also absent is the interaction between the backbone amide nitrogen of Arg195 and the γ phosphate. Thus, the γ phosphate appears to be missing two critical contacts. Finally, the amino nitrogen of Asn111 forms a hydrogen bond with the γ phosphate oxygen, therefore restraining the γ phosphate out of the P-loop cavity. In apo FtsY Arg195 is positioned away from the phosphate-binding cavity. The preceding glycine, Gly194, forms a salt bridge with the P-loop and orients the backbone nitrogens away from the γ phosphate. In F1, the sidechain of Arg195 moves into the active site such that it coordinates a water molecule with the γ phosphate oxygen and Asn111 sidechain from the P-loop. The amido group of Asn111 also forms a hydrogen bond with the γ phosphate oxygen. These interactions act to force the γ-phosphate out of the binding pocket and constrict the P-loop. The measured distance between the Cα of P-loop residues Asn111 and Thr116 is 9.8 Å in F1. This constriction is not as great as seen in the Ffh-NG·GMPPNP complex [10] (∼8.8 Å) but still represents a constriction of 0.7 Å as compared to FtsY in complex with Ffh-NG.

## Discussion

The structures of FtsY described here represent snapshots of FtsY and nucleotide in various active site conformations. We interpret these structures as representing discrete states along the pathway of activation and dissociation of FtsY in the targeting cycle. These structures can also be compared with the product complex of FtsY with GDP bound [11]. The latter structure crystallizes in a different space group (I4) from the structures reported here, reflecting the conformational change upon going from the substrate bound, to product bound conformations. This step-wise activation potentially provides specific regulatory points along the protein-targeting pathway.

### Structural Basis for Nucleotide Promiscuity Revealed

**Figure 4:**
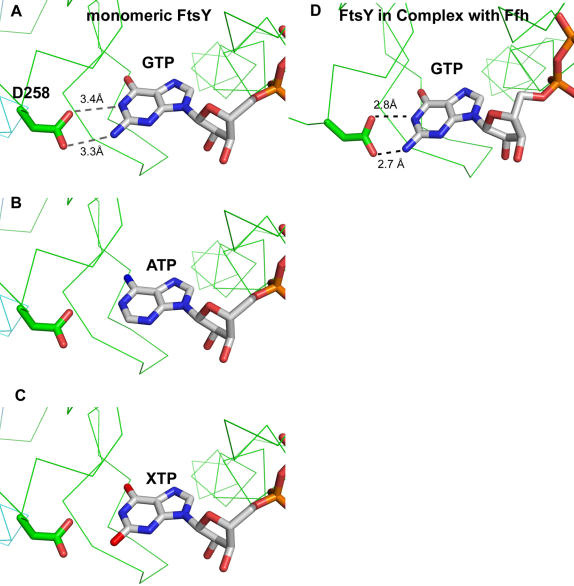
SRP-induced nucleotide specificity of FtsY. The specificity-determining hydrogen bonding interactions between GMPPNP and Asp258 are on average 0.6 Å more distant in the (A) monomeric nucleotide bound form of FtsY (F1) than for (D) FtsY in complex with Ffh. This additional spacing allows for the non-specific binding and hydrolysis of (B) ATP and (C) XTP in the monomeric form but not the complexed form of FtsY as observed in biochemical studies by Shan and Walter [24]. The ATP and XTP molecules were modeled based on the position of GMPPNP in the monomeric form of FtsY (F1).

The most obvious result in our study relates the variation in the position of conserved motif IV with a structural explanation for the poor base specificity and promiscuous nucleotide hydrolysis reported in nucleotide binding studies of FtsY by Shan and Walter [24]. In solution, FtsY displays little specific affinity for GTP versus ATP, XTP and other nucleotides. However, complex formation with Ffh increases nucleotide specificity 10^−3^-fold [15]. An examination of the coordinating distance of motif IV aspartate to the guanine ring provides a structural account for these observations. The coordination distance observed between Asp258 and the guanine nucleotide is most optimal in the complex with Ffh yet dissipates in the absence of Ffh as observed in F1. In addition, F2 shows bound nucleotide despite no coordination from Asp258 (Figure 4). This is a simple structural explanation for the low specific affinity.

### Nucleotide Binding Alone does not Initiate the Catalytic Cycle of the Targeting Complex

The conserved structural elements in F1 and further in F2 suggest a sequential ordering of the active site during complex formation. The increasing coordination between Asp258 and GMPPNP from apo FtsY to F2 and further in F1 relates to a closing of the active site and an increase in the coordination of the guanine base of the nucleotide. Complex formation with Ffh-NG directs FtsY IBD residues Arg142 and Gln148 to interact with the β- and γ-phosphate groups while catalytic Asp139 activates the proposed nucleophilic water. In addition, Asn111 forms the only sidechain contact with the *trans* substrate of Ffh-NG, a hydrogen bond with the 3′O of the GMPPCP. Analogous conserved residues act in the same manner in the Ffh-NG component of the complex. Further, the nucleotides themselves act *in trans* to stabilize the negative change on the γ-phosphate formed during GTP hydrolysis in contrast to most other GTPases that require an external activation residue from their respective GAPs. Both F1 and F2 show a total disruption in these interactions and in fact exhibit a coordination that positions the γ-phosphate out of the active site. Without these intra- and interactions, hydrolysis of GTP is not favored.

### N-terminal Helix Represses GTPase Activity

**Figure 5:**
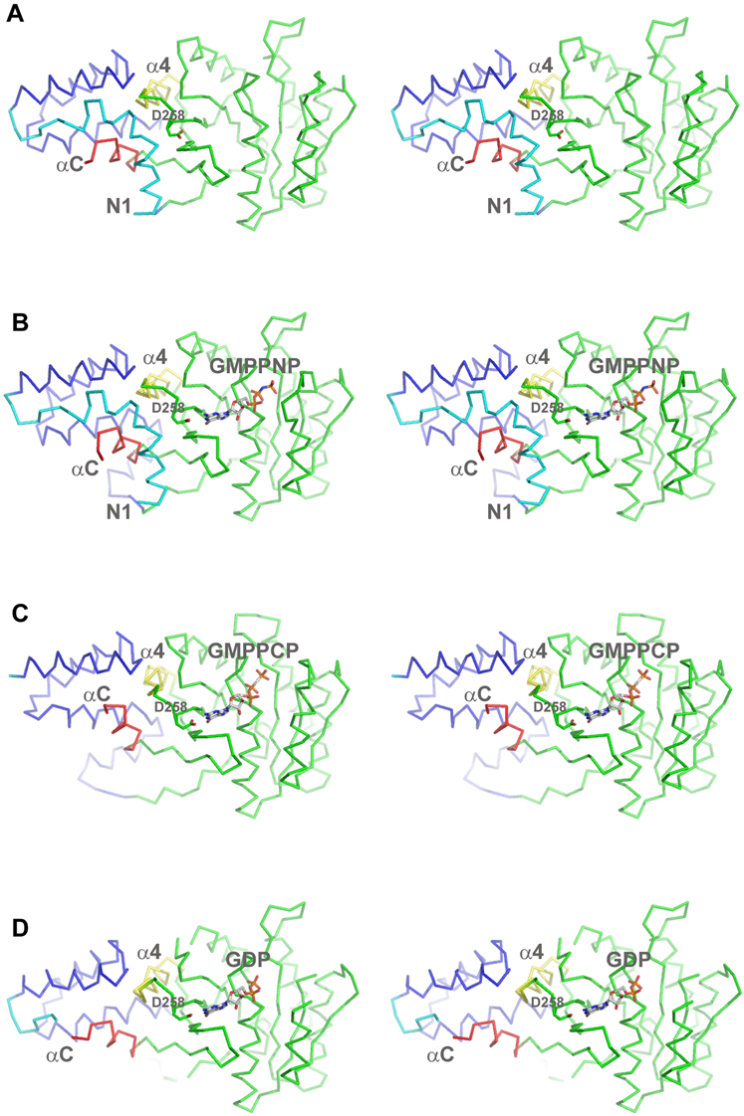
The N-terminal helix N1 packs against the nucleotide-specifying motif IV. The structures of FtsY in (A) apo form, (B) monomeric GMPPNP-bound form, (C) GMPPCP-bound form from the targeting complex with Ffh, and (D) monomeric GDP-bound form were aligned based on the P-loop; the N-domain (blue) and G-domain (green) are highlighted. In the structures of apo and GMPPNP-bound FtsY the N-terminal helix, N1 (cyan), extends into the N-domain and packs against the conserved GTPase motif IV which positions the nucleotide-specifying Asp258 to interact with the nucleotide. The α4 helix (yellow) anchors the interface between the N and G domains. The C-terminal helix, αC (red), which along with N1 pack together at the N/G interface, is observed in a similar arrangement in the three monomeric forms (A, B, D) but rearranges in the complex form of FtsY (C).

**Figure 6:**
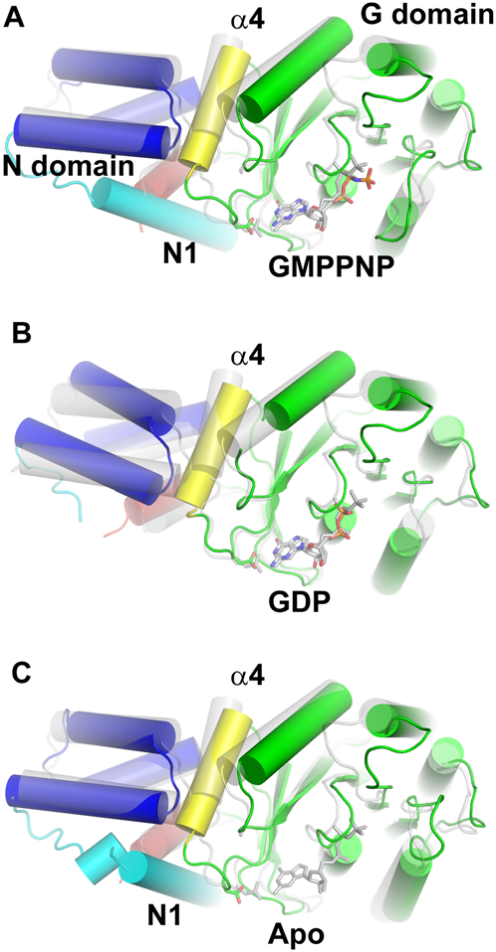
N1 mediates domain shift between the N and G domains. (A) FtsY:GMPPNP, (B) FtsY:GDP, and (C) apo FtsY superimposed with FtsY:GMPPCP from the targeting complex with Ffh (transparent grey) (alignment on P-loop as in Figure 5). The N/G domain organization is similar for the apo, monomeric GMPPNP-bound form and complex form of FtsY, but differs in the GDP structure. Deletion of the N1 helix (cyan) in the GDP structure allows for this observed shift in the N-domain (blue) relative to the G-domain (green).

The A-domain is an N-terminal domain which has been proposed to anchor FtsY to the membrane [25], [26] although it is not essential for SRP-mediated targeting [27]. *T.aquaticus* FtsY lacks the A-domain, but retains an N-terminal helix, termed N1. The N1 helix becomes susceptible to proteolysis upon formation of targeting complex with Ffh [28] and deletion of N1 causes a ∼4–5 fold increase in GTPase activity. In contrast to the structures presented here, the N1-deleted form of FtsY from *T. aquaticus* binds GDP in a canonical manner including the specificity-determining hydrogen bonding interactions with Asp258 [11]. However, deletion of N1 has a pronounced affect on the position of the N-domain relative to the G-domain upon nucleotide binding when compared to either apo FtsY, FtsY:GMPPNP or FtsY:GMPPCP from the targeting complex with Ffh (Figure 5 and Figure 6). It is also important to note that the C-terminal helix (αC) assumes a similar position in the apo, GMPPNP and the GDP structures of FtsY, but rearranges upon complex formation with Ffh. Given (*i*) the proteolytic susceptibility of N1 upon complex formation, (*ii*) increase in GTPase activity upon deletion of N1 and (*iii*) the observation of a canonical active site for monomeric FtsY:GDP, the N1 helix might act to repress GTPase activity in the monomeric form of FtsY. This could be accomplished by favoring an N/G domain organization that favors a more open active site. Upon complex formation, N1 rearranges for proper complex formation and active site optimization. In this manner, the N1 helix would act as a negative regulatory element; maintaining low activity in the non-targeting, monomeric form of FtsY as observed in the structures presented in this paper. This regulation might also be affected by interactions with the membrane and membrane-bound translocon.

### Step-Wise Activation and Dissociation of the SRP-Mediated Targeting Complex

GTP-hydrolysis by GTPases is intrinsically slow but can be accelerated by binding of specific external factors. SRP GTPases are unique in that activation of GTP-hydrolysis occurs upon formation of a complex of two GTPases. Our data support and expand an emerging model that explains some of the complexity in the catalytic cycle of FtsY and Ffh. SRP GTPases are regulated not at the point of nucleotide binding or exchange, but rather through a ‘step-wise’ mechanism. FtsY and Ffh bind GTP in an “open” or “primed” state. An “active” conformational state is reached upon the concerted rearrangements of the SRP GTPase conserved sequence elements after complex formation with SRP. A concerted step-wise formation would also imply that nucleotide hydrolysis and release might also be step-wise. Nucleotide release in GTPases is typically regulated by guanine nucleotide exchange factors by disruption of Mg^2+^ coordination, perhaps by displacement of Mg^2+^ from the active site [29]–[31] . It may be that the IBD gates coordination of the active site Mg^2+^ and in turn contributes to the low affinity for nucleotide through disruption of proper coordination architecture in the monomer form. The N1 helix might also contribute to regulation of the GTPase cycle and promote a step-wise activation by repressing GTPase activity in the monomeric form. The FtsY GMPPNP structures described here depict conformational states along the activation pathway for SRP GTPases in the targeting reaction and reveal the structural basis for the futile nature of GTP hydrolysis in monomeric FtsY. In addition, these structures provide insight into the step-wise ability of FtsY to self-regulate its GTPase activity as well as reset to the apo state through release of the nucleotide for subsequent rounds of targeting.

## Materials and Methods

### Crystallization

*T. aquaticus* FtsY was expressed in *E. coli* strain BL21 (DE3) (Novagen) using a pET21 plasmid and purified to homogeneity. FtsY was concentrated to 9 mg ml^−1^ in 50 mM Hepes pH 7.5, 150 mM potassium acetate pH 7.5, 2.5mM magnesium acetate and 2 mM β-OG, octyl-β-d-glucoside. Ffh was prepared as previously described [7]. Crystallization attempts of FtsY and Ffh with 3x molar excess of GMPPNP, and 10mM MgCl_2_ were carried out as hanging-drops with the vapor diffusion method at room temperature and crystals initially grew out of two conditions: 2.0 M ammonium sulfate and 2.0 M ammonium sulfate, 100 mM sodium acetate. Identification of FtsY as the sole component of the crystals was evaluated by gel electrophoresis and mass spectrometry. Additional crystals were grown from 18% PEG 2000, Tris pH 7.5, 100mM NaCl . For MAD, a mercury derivative of FtsY was used. Purified FtsY was incubated with 10 mM TCEP-HCl (Tris(2-Carboxyethyl) Phosphine, Hydrochloride) for 4 hours to reduce oxidation on the cysteine (Cys136). Following buffer exchange with a Hepes buffer to eliminate reducing agent, 10mM of the Hg compound CMNP (6-chloromercuri 2,4-dinitrophenol-Kodak) was added to the protein and incubated for 4 hours. The FtsY-Hg complex was then buffer exchanged with Hepes buffer using Centricon YM-10K (Amicon) to eliminate unbound Hg. The covalent binding of Hg was confirmed with mass spectrometry.

### Data collection

Data collection parameters and statistics for all data are listed in Table 1. FtsY-Hg crystals diffracted to 1.8 Å and a three wavelength MAD data set was collected at the Advance Light Source in Berkeley (ALS) beamline 5.0.2. Data were measured at three wavelengths, λ_f″_ = 1.006 Å, λ_f′_ = 1.009 Å and λ_high remote_ = 0.993 Å. Data collected at SSRL were integrated and reduced with MOSFLM and SCALA [32] and data collected at ALS were processed with HKL2000 [33]. All reduced data were truncated and placed on an absolute scale with Wilson B-factor estimation with TRUNCATE from the CCP4 program suite [32].

### Structure determination

Two mercury sites were resolved by hand and confirmed using an automated heavy atom search procedure in CNS [34]. Heavy atom refinement and phasing was done with SHARP and resulted in an overall FOM of 0.51 for MAD and 0.75 for combined SIR and MAD. Iterative rounds of solvent flattening, NCS averaging, and phase combination of SIR and MAD phases were calculated with the CCP4 program DMmulti. The calculated phases were used in phased molecular replacement to position a poly-alanine version (loops omitted) of the previously determined *E. coli* FtsY structure into the electron density. Initial density in the active site from the MAD phased maps as well as simulated annealing omit maps calculated from the molecular replacement model showed clear density for the tri-phosphate nucleotide. Figures were generated using SPDBV and PyMol.

## References

[1] Walter P, Johnson AE (1994). Signal sequence recognition and protein targeting to the endoplasmic reticulum membrane.. Annu Rev Cell Biol.

[2] Bourne HR, Sanders DA, McCormick F (1991). The GTPase superfamily: conserved structure and molecular mechanism.. Nature.

[3] Keenan RJ, Freymann DM, Stroud RM, Walter P (2001). The Signal Recognition Particle.. Annu Rev Biochem.

[4] Jagath JR, Rodnina MV, Wintermeyer W (2000). Conformational changes in the bacterial SRP receptor FtsY upon binding of guanine nucleotides and SRP.. Journal of Molecular Biology.

[5] Moser C, Mol O, Goody RS, Sinning I (1997). The signal recognition particle receptor of Escherichia coli (FtsY) has a nucleotide exchange factor built into the GTPase domain.. Proc Natl Acad Sci U S A.

[6] Powers T, Walter P (1995). Reciprocal Stimulation of GTP Hydrolysis by Two Directly Interacting GTPases.. Science.

[7] Freymann DM, Keenan RJ, Stroud RM, Walter P (1997). Structure of the conserved GTPase domain of the signal recognition particle.. Nature.

[8] Montoya G, Svensson C, Luirink J, Sinning I (1997). Expression, crystallization and preliminary X-ray diffraction study of FtsY, the docking protein of the signal recognition particle of E. coli.. Proteins.

[9] Freymann DM, Keenan RJ, Stroud RM, Walter P (1999). Functional changes in the structure of the SRP GTPase on binding GDP and Mg2+GDP.. Nature Structural Biology.

[10] Padmanabhan S, Freymann DM (2001). The conformation of bound gmppnp suggests a mechanism for gating the active site of the srp gtpase.. Structure (Camb).

[11] Gawronski-Salerno J, Coon VJ, Focia PJ, Freymann DM (2006). X-ray structure of the T. Aquaticus Ftsy:GDP complex suggests functional roles for the C-terminal helix of the SRP GTPases.. Proteins.

[12] Montoya G, Svensson C, Luirink J, Sinning I (1997). Crystal structure of the NG domain from the signal-recognition particle receptor FtsY.. Nature.

[13] Gariani T, Samuelsson T, Sauer-Eriksson AE (2006). Conformational variability of the GTPase domain of the signal recognition particle receptor FtsY.. J Struct Biol.

[14] Joint Center for Structural Genomics (2007).

[15] Peluso P, Shan SO, Nock S, Herschlag D, Walter P (2001). Role of SRP RNA in the GTPase cycles of Ffh and FtsY.. Biochemistry.

[16] Shan S, Walter P (2003). Induced nucleotide specificity in a GTPase.. Proceedings of the National Academy of Sciences of the United States of America.

[17] Egea PF, Shan SO, Napetschnig J, Savage DF, Walter P (2004). Substrate twinning activates the signal recognition particle and its receptor.. Nature.

[18] Focia PJ, Shepotinovskaya IV, Seidler JA, Freymann DM (2004). Heterodimeric GTPase core of the SRP targeting complex.. Science.

[19] Gawronski-Salerno J, Freymann DM (2006). Structure of the GMPPNP-stabilized NG domain complex of the SRP GTPases Ffh and FtsY.. J Struct Biol.

[20] Focia PJ, Gawronski-Salerno J, Coon JS, Freymann DM (2006). Structure of a GDP:AlF4 complex of the SRP GTPases Ffh and FtsY, and identification of a peripheral nucleotide interaction site.. J Mol Biol.

[21] Connolly T, Gilmore R (1989). The signal recognition particle receptor mediates the GTP-dependent displacement of SRP from the signal sequence of the nascent polypeptide.. Cell.

[22] Connolly T, Rapiejko PJ, Gilmore R (1991). Requirement of GTP hydrolysis for dissociation of the signal recognition particle from its receptor.. Science.

[23] Shan SO, Stroud RM, Walter P (2004). Mechanism of association and reciprocal activation of two GTPases.. PLoS Biol.

[24] Shan SO, Walter P (2003). Induced nucleotide specificity in a GTPase.. Proc Natl Acad Sci U S A.

[25] de Leeuw E, Poland D, Mol O, Sinning I, ten Hagen-Jongman CM (1997). Membrane association of FtsY, the E. coli SRP receptor.. FEBS Lett.

[26] de Leeuw E, te Kaat K, Moser C, Menestrina G, Demel R (2000). Anionic phospholipids are involved in membrane association of FtsY and stimulate its GTPase activity.. Embo J.

[27] Zelazny A, Seluanov A, Cooper A, Bibi E (1997). The NG domain of the prokaryotic signal recognition particle receptor, FtsY, is fully functional when fused to an unrelated integral membrane polypeptide.. Proceedings of the National Academy of Sciences of the United States of America.

[28] Shepotinovskaya IV, Freymann DM (2002). Conformational change of the N-domain on formation of the complex between the GTPase domains of Thermus aquaticus Ffh and FtsY.. Biochim Biophys Acta.

[29] Sprang SR (1997). G protein mechanisms: insights from structural analysis.. Annu Rev Biochem.

[30] Zhang JT (2000). Determinant of the extracellular location of the N-terminus of human multidrug-resistance-associated protein.. Biochemical Journal.

[31] Ihara K, Muraguchi S, Kato M, Shimizu T, Shirakawa M (1998). Crystal structure of human RhoA in a dominantly active form complexed with a GTP analogue.. J Biol Chem.

[32] CCP4 (1994). The CCP4 Suite: Programs for Protein Crystallography.. Acta Crystallogr D.

[33] Otwinowski Z, Minor W (1997). Processing of X-ray Diffraction Data Collected in Oscillation Mode.. Methods in Enzymology.

[34] Brunger AT, Adams PD, Clore GM, DeLano WL, Gros P (1998). Crystallography & NMR system: A new software suite for macromolecular structure determination.. Acta Crystallogr D Biol Crystallogr.

